# The Influence of Regional Distribution and Pharmacologic Specificity of GABA_A_R Subtype Expression on Anesthesia and Emergence

**DOI:** 10.3389/fnsys.2017.00058

**Published:** 2017-08-22

**Authors:** Iris Speigel, Edyta K. Bichler, Paul S. García

**Affiliations:** ^1^Department of Anesthesiology, Emory University School of Medicine, Atlanta GA, United States; ^2^Research Division, Atlanta Veteran’s Affairs Medical Center, Atlanta GA, United States

**Keywords:** GABA, receptor trafficking, surface expression, extra-synaptic receptors, tonic inhibition, anesthesia, POCD

## Abstract

Anesthetics produce unconsciousness by modulating ion channels that control neuronal excitability. Research has shown that specific GABA_A_ receptor (GABA_A_R) subtypes in particular regions of the central nervous system contribute to different hyperpolarizing conductances, and behaviorally to distinct components of the anesthetized state. The expression of these receptors on the neuron cell surface, and thus the strength of inhibitory neurotransmission, is dynamically regulated by intracellular trafficking mechanisms. Pharmacologic or activity-based perturbations to these regulatory systems have been implicated in pathology of several neurological conditions, and can alter the individual response to anesthesia. Furthermore, studies are beginning to uncover how anesthetic exposure itself elicits enduring changes in subcellular physiology, including the processes that regulate ion channel trafficking. Here, we review the mechanisms that determine GABA_A_R surface expression, and elaborate on influences germane to anesthesia and emergence. We address known trafficking differences between the intrasynaptic receptors that mediate phasic current and the extra-synaptic receptors mediating tonic current. We also describe neurophysiologic consequences and network-level abnormalities in brain function that result from receptor trafficking aberrations. We hypothesize that the relationship between commonly used anesthetic agents and GABA_A_R surface expression has direct consequences on mature functioning neural networks and by extension ultimately influence the outcome of patients that undergo general anesthesia. Rational design of new anesthetics, anesthetic techniques, EEG-based monitoring strategies, or emergence treatments will need to take these effects into consideration.

## Introduction

Anesthetics generate the most recognizable feature of general anesthesia, unconsciousness, by modulating ion channels in the central nervous system, especially the GABA_A_ receptor (GABA_A_R) (Garcia et al., 2010). The exposure of GABA_A_Rs to neurotransmitters and modulators (e.g., benzodiazepines and anesthetics) depends on the number and distribution of receptors existing on the cell surface in a particular brain region. In addition to abundance, variation is also an important determinant of GABAergic signaling. The most prominent classifying features are receptor proximity to the synapse and the type of current it mediates, with subtypes broadly characterized as “intra-synaptic” receptors mediating phasic currents or “extra-synaptic” receptors mediating tonic currents. Mutagenic and recombinant studies have demonstrated that these subtypes have profound intrinsic differences in sensitivity to GABA, pharmacologic modulation, and anesthetic agents. These subtype-specific interactions are critical to understanding how modulation of inhibitory neurotransmission is achieved under clinical or diseased conditions, and to interpreting the basis for neuron ensemble activity patterns that manifest during anesthesia, e.g., those reflected by electroencephalograph (EEG) recordings. The clinical implication is that subtle differences in the CNS distribution or expression levels of certain GABA_A_R subtypes could underlie phenotypic differences in sensitivity to anesthesia. These differences might manifest as problems related to appropriate dosing of anesthetic drugs (i.e., as an increased risk of awareness with recall) during surgery or as an enhanced sensitivity to the hypoactivity commonly encountered in the immediate post-anesthesia period, emergence. Moreover, increased functional knowledge of region-specific as well as subcellular localization of GABA_A_R subtypes may hold the key to understanding the neurological consequences of anesthesia, e.g., emergence complications, delirium, or long-term cognitive dysfunction.

Many drugs which modulate GABA_A_R channel function also change surface expression of GABA_A_Rs, e.g., flumazenil (Kuver and Smith, 2016), alcohol (Gonzalez et al., 2012), and neurosteroids (Abramian et al., 2014). Interestingly, recent studies demonstrate that anesthetic agents may as well (Zurek et al., 2014; Li et al., 2015). This review will describe pharmacologic and activity based influences on GABA_A_R surface expression and its relationship to anesthesia and emergence. We will review what is known (and what is unknown) regarding brain region specificity of GABA_A_R subtypes and how the GABA_A_R landscape might change during surgical anesthesia. Then, we will outline how GABA_A_R surface expression is dynamically maintained by trafficking between subcellular compartments, and specify where subtype variability imparts important functional and trafficking properties. We will also illustrate how subtype-specific surface expression underlies inhibitory signaling and how subtype heterogeneity underlies the behavioral endpoints of anesthesia. Finally, we discuss how trafficking changes can alter clinical sensitivity to anesthesia, and how trafficking phenotypes may contribute to complications in the recovery of cognitive functions from anesthesia.

## Modulation of GABA_A_Rs in the CNS by Anesthetics

Physiologically, drugs that produce general anesthesia for use in surgery cause a progressive depression of electrochemical communication among cells in the central nervous system. The cardinal target of anesthesia, consciousness, is supported by a network of arousal centers residing in subcortical nuclei. These excitatory projections connect to each other and to the rest of the brain. In turn, these nuclei receive inhibitory projection from the hypothalamus (preoptic area), the thalamus, and the cortex (Silber and Rye, 2001). The predominate explanation for the process of initiating unconsciousness is that when anesthetic agents infuse the brain during induction, this network of interdependent excitatory/inhibitory connections is disturbed, and the arousal nuclei decrease neurotransmitter output (Brown et al., 2011). Ultimately, the diminished excitatory drive onto pyramidal neurons of the cortex and thalamus causes network activity to collapse, leading to a decrease in cortical activity, energy, and integration of information – ultimately a state of quiescence (Mashour et al., 2005; Alkire et al., 2008; Brown et al., 2011).

While they primarily generate unconsciousness (and other endpoints of anesthesia) by actions on ion channels in the central nervous system, an important caveat to this paradigm is that anesthetics have molecular targets on many other proteins in the body (Pan et al., 2008). In neurons, affected processes include cellular housekeeping systems such as mitochondrial transition, calcium homeostasis, and actin polymerization (Platholi et al., 2014; Vutskits and Xie, 2016). “Off-target” effects can underlie catastrophic patient reactions, such as the hypersensitivity of skeletal muscle ryanodine receptor channel function to volatile agents in malignant hyperthermia (Correia et al., 2012). Subtler effects with latent complications are suspected in the development of several neurodegenerative diseases, namely Alzheimers’ via β-Amyloid (Xie and Xu, 2013). Later, we review anesthetic-protein interactions in the second messenger signaling protein pathways known to control GABA_A_R trafficking. Of non-ion channel proteins, there are no obvious structural motifs of the intracellular protein targets of anesthetics.

## GABA_A_R Diversity and Distribution

The crystallized GABA_A_R structure was reported in 2014 (Miller and Aricescu, 2014). GABA_A_Rs are related by protein homology to of the Cys-loop superfamily of ligand-gated ion channels, which share similarities with the prokaryotic gleobacter ligand gated ion channel (Nury et al., 2011). GABA_A_Rs assemble into heteropentamers from 19 different possible subunits that are classified by protein sequence homology as α1–6, β1–3, γ1–3, δ, ε, θ, or ρ1–2. Most naturally occurring receptors contain two α, two β, and either a γ or δ subunit, with α1β2γ2 being the most abundant subtype in the mammalian CNS (McKernan and Whiting, 1996). Only nine different configurations are unequivocally expressed in abundance in the mammalian brain (Olsen and Sieghart, 2008). **Table 1** describes some of the known research of these configurations. Individual subunit combinations are unique with respect to their pharmacology (Olsen and Sieghart, 2008), distribution among different brain regions (Wisden and Seeburg, 1992), cell-type specific expression (Lee and Maguire, 2014), and subcellular localization (Mody and Pearce, 2004).

**Table 1:** GABA_A_R subtype diversity and anatomical distribution.

GABA_A_R	I/E	Pharmacology	Anatomic region	Cell type	Reference
α1βxγ2	Intra	Agonist: zolpidem	Basal forebrain, PPT, LDT	Cholinergic neurons	Winsky-Sommerer, 2009
		Sedative, hypnotic	Cortex (Layers 1–6)	Interneurons, pyramidal cells	Wisden et al., 1992
			Cerebellum	Purkinje, granule cells	Jechlinger et al., 1998; Nusser et al., 1998
			Hippocampus (CA1–3)	Interneurons, pyramidal cells	Gao and Fritschy, 1994
			Hippocampus (DG)	Interneurons, granule cells	Gao and Fritschy, 1994
			Thalamus (VB)	Thalamocortical relay neurons	Browne et al., 2001
			TMN	Histaminergic cells	May et al., 2013

α2βxγ2	Intra	Agonist: SL651498	Cortex (Layers 1–4)	Pyramidal cells	Nusser et al., 1996
		Anxiolytic sedative	Hippocampus (CA1–3)	Pyramidal cells	Sperk et al., 1997
			Spinal cord	Primary afferents, intrinsic neurons	Bohlhalter et al., 1996; Paul et al., 2012
			Dorsal horn		
			TMN	Histaminergic cells	May et al., 2013

α3βxγ2	Intra	Agonist: TPA-023	Spinal cord	Primary afferents, intrinsic neurons	Bohlhalter et al., 1996; Paul et al., 2012
		Non-sedating anxiolysis and muscle relaxation	Dorsal horn		
			Locus coeruleus	Noradrenergic neurons	Winsky-Sommerer, 2009
			Raphe nucleus	Serotonergic neurons	Winsky-Sommerer, 2009
			Thalamus (RTN)	Inhibitory neurons	Browne et al., 2001

α4β3δ	Extra	BZD insensitive	Cortex (Layers 2/3)	Pyramidal cells	Huang et al., 2015
		Neurosteroid sensitive	Hippocampus (DG)	Granule cells	Mortensen and Smart, 2006
			Thalamus (VB)	Thalamocortical relay neurons	Jia et al., 2005

α4β2γ2	Extra	Weak basal expression, upregulated by ethanol	Hippocampus (DG)	Granule cells	Liang et al., 2007
			Thalamus (VB)	Thalamocortical relay neurons	Sur et al., 1999a

α5β3γ2	Extra	High VGA sensitivity, amnesia	Hippocampus (CA1–3)	Pyramidal cells	Caraiscos et al., 2004a
			Cortex (Layer 5)	Pyramidal cells	Yamada et al., 2007

α6βxγ2	Intra	Anesthetic ataxia BZD insensitive	Cerebellum	Granule cells	Jechlinger et al., 1998; Nusser et al., 1998
α6βx2δ	Extra				


*The main intrasynaptic receptor for most regions is α1β2γ2, the most abundant subtype in the brain. In contrast, the extra-synaptic receptor subtype that mediates tonic current varies the most between brain regions. The thalamus, hippocampus, and cerebellum predominantly use α4βxδ, α5β3γ2, and α6βxδ extra-synaptic receptors, respectively. Generally, the α4 and α6 containing receptors lack benzodiazepine sensitivity, whereas the γ2 containing receptors retain sensitivity. With regard to anesthesia, the α4–6 receptors display enhanced sensitivity to volatile gas anesthetics. BZD, benzodiazepine; DG, dentate gyrus; LDT, lateral dorsal tegmental nucleus; PPT, pedunculopontine tegmental nucleus; RTN, reticular nucleus of the thalamus; TMN, tuberomammillary nuclei; VB, ventrobasal nucleus of the thalamus; VGA, volatile gas anesthetic.*

For all these receptors, the primary endogenous ligand is GABA, which gates the chloride-permeable ion channel pore. Canonically, GABA-ergic inhibition curtails neuronal activity. This effect occurs via either hyperpolarization or shunting, depending on the chloride driving force, the resting membrane potential, and the action potential threshold at the time the impulse arrives. GABA is depolarizing in the earliest developing neurons that have not yet expressed specific membrane transporters to establish the usual chloride gradient (Ganguly et al., 2001; Ben-Ari et al., 2007; Ben-Ari, 2015). Because the neonate brain contains neurons at many different stages of development, anesthetics potentiating GABA-mediated currents could be excitatory in some neurons.

At clinical doses, most anesthetics act as positive allosteric modulators, increasing channel function by binding to protein cavities remote from the ligand binding site and causing conformational changes in structure that increase and prolong the response to GABA. Several interaction sites for volatile agents on the GABA_A_R have been identified through mutagenic and biophysical experiments (Franks, 2006). Similarly, sites for intravenous agents have been identified (Miller and Aricescu, 2014; Weiser et al., 2014). On the intracellular face of the receptor, each subunit contains a small cytoplasmic loop between the first and second transmembrane domains and a larger cytoplasmic loop between the third and fourth transmembrane domain. This larger loop domain has the largest sequence variability between subtypes (Olsen and Sieghart, 2008), and contains several sites for protein–protein interactions. This intracellular loop is the main site for regulatory control of membrane trafficking by intracellular signaling pathways.

Receptor sequence heterogeneity is well studied as a source of subtle biophysical properties that lead to pharmacologic specificity for various endogenous and exogenous substances, including anesthetic agents. Recombinant studies demonstrate that the α subunit contributes to biophysical difference in GABA sensitivity, as heterologously expressed α1β2γ2 receptors show a lower affinity than α4β2γ2 (Jia et al., 2005). In most of the brain, intrasynaptic receptors contain α1–3 whereas extra-synaptic receptors contain α4–6 (Farrant and Nusser, 2005). Although all of these subunits are subject to allosteric modulation by anesthetic agents (with the notable exception of ρ), the sensitivity to anesthetics is not equal, and the extrasynaptic receptors are comparatively sensitive to modulation by anesthetics (Bonin and Orser, 2008).

## Intrasynaptic and Extra-Synaptic GABA_A_R Populations Generate Phasic and Tonic Current

GABA-ergic neurotransmission evokes two distinct forms of inhibitory currents. The basis for these two types of currents lies within receptor subunit composition and subcellular location. Phasic currents are primarily mediated by low-affinity intrasynaptic receptors, which generate brief and quickly desensitizing inhibitory post-synaptic currents (IPSCs) in response to synaptic neurotransmission (Mody and Pearce, 2004). Intrasynaptic receptor clustering is based on the construction of the GABAergic synapse, which is made of a post-synaptic density of several proteins organized opposite to the presynaptic terminal (Tretter et al., 2012). Intrasynaptic receptors are typically are made of large clusters of α1, α2, or α3 containing receptors anchored to the post-synaptic cytoskeleton by the scaffold protein gephyrin. Inside the synapse, these receptor clusters are transiently exposed to high (∼mM) concentrations of GABA from synaptic vesicular release, and generate phasic currents that terminate after GABA is cleared from the synapse (Stell and Mody, 2002). Even before clearance by glial or neuronal pumps, phasic currents rapidly desensitize generating the characteristic decayed shape of the ISPC.

Tonic currents are mediated by extra-synaptically located receptors, which generate persistent conductances in response to smaller (∼nM–μM) amounts of GABA existing in the intercellular space (Stell and Mody, 2002). The extra-synaptic receptors have a higher affinity for GABA and ideal for detection of small amounts of neurotransmitter. Additionally, these receptors exhibit little or no accommodation to saturating concentrations of GABA, a phenomenon known as desensitization. The GABA that evokes tonic current is thought to arise from two overlapping sources, ambient and spill-over GABA. Ambient GABA describes the minute amount preexisting in the extracellular space. Measurements from *in vivo* microdialysis suggest peri-synaptic GABA is between ≈30–300 nM, depending on brain region (Lerma et al., 1986; Tossman et al., 1986). Notably, GABA levels can change with physiological and behavioral states (de Groote and Linthorst, 2007). Spill-over GABA is released by synaptic transmission and diffuses away to have paracrine action on remote receptors, e.g., on other neurons. These terms have conceptual overlap and do not demarcate two types of GABA, but rather distinguish effects in spatiotemporal relation to synaptic transmission. High levels of phasic inhibition can drive spillover and raise local ambient GABA. For example, an increase in inhibitory activity can encourage sleepiness in part due to the accumulation of ambient GABA.

Comparatively, tonic and phasic inhibitory currents generate different effects on neuronal excitability and consequent inhibitory/excitatory signaling dynamics (Mody and Pearce, 2004). Functionally, phasic GABA currents decrease the instantaneous probability of action potential firing. In contrast, tonic currents cause a more persistent ongoing inhibition or hyperpolarization that is not temporally discrete. Because tonic current decreases membrane resistance, hypothetically the behavior of more actively firing neuron would be more sensitive to tonic inhibition than a less actively firing one. How these conductances ultimately affect individual neurophysiological process depends on cell-type specific intrinsic properties as well as circuit-specific properties. For example, interneuron spiking behavior changes dramatically with the addition of tonic GABA inhibitory input (Pavlov et al., 2014) in a bidirectional manner related to intrinsic cellular properties. Subcellular innervation patterns onto target neurons (e.g., axo-axonic vs. axo-somatic vs. axo-dendritic) would also critically determine post-synaptic effect. Further complicating a straightforward interpretation of anesthetic effects as a function of GABAergic modulation is the widespread expression of GABA_A_Rs. An inhibitory input onto a target neurons could be modulated at the post-synaptic level (anesthetic enhancement of post-synaptic GABA_A_Rs), or at the pre-synaptic level (anesthetic enhancement of interneuron GABA_A_Rs and depressed output and disinhibiting of the target neuron). In neuroscience at large, recent significant technical advances in methods of isolating cell-type specific actions via genetically select identification and activation have revolutionized empirical characterization of neural circuits (Murphey et al., 2014). The application of similar strategies to the study of anesthetic mechanisms could clarify with greater precision how anesthetics affect inhibitory actions within select neural circuits.

Finally, phasic and tonic conductances also shape emergent neural network properties such as oscillations (Mann and Mody, 2010). The temporal dynamics of phasic inhibition are important because synchronizing population activity into slow oscillatory waves requires precise timing of inhibitory input. Tonic current is important in rhythmogenesis as an input counteracting tonic excitation, as well as factor in the activation of hyperpolarization-gated conductances.

## Subunit Composition and Sensitivity to Anesthesia-Related Drugs

At clinically used doses, GABA-ergic anesthetics generally potentiate phasic currents by prolonging IPSC duration, and tonic currents by increasing the amplitude and charge transfer. Pharmacologic substances do not affect tonic and phasic conductances in the same way (Bieda et al., 2009). An enhanced sensitivity to GABA-ergic anesthetic drugs is a common feature of extra-synaptic receptors (Bonin and Orser, 2008). In hippocampal pyramidal excitatory neurons, low concentrations (25 μM) of isoflurane enhance the α5 mediated tonic but not phasic current (Caraiscos et al., 2004b). In the same experiments, 100 μM is the minimal dose to enhance IPSCs. These reactions to sub-anesthetic doses may explain why low doses of our anesthetics produce a sedation phenotype. Similarly, direct activation-low doses of isoflurane will also directly activate α4s, whereas only high doses can directly activate α1 (Raines et al., 2003). For reference a typical concentration of isoflurane which might mediate unconsciousness clinically is 250–300 μM (Hemmings and Egan, 2013).

The behavioral responses of transgenic mice with mutated extra-synaptic subunits suggest that disabling the receptors mediating tonic current is sufficient to abolish specific elements of anesthesia. For example, deleting the α4 subunit significantly reduces the effect of isoflurane on amnesia (context-dependent fear learning) with no effect on hypnosis or immobility (Rau et al., 2009). The potential importance of this is that intraoperative amnesia likely happens because low sub-anesthetic doses of isoflurane are sufficient to impair learning and memory by increasing α4βxδ tonic current in the dentate gyrus, so changes in α4 surface expression could affect amnesia without affecting the other anesthetic endpoints that are more salient during operation.

Benzodiazepines (e.g., midazolam) allosterically potentiate receptors containing the γ2 subunit, with sensitivity determined by the α subunit (Jenkins et al., 2001). Conversely, the δ containing receptors which mediate tonic current are insensitive to benzodiazepines but very sensitive to allosteric enhancement by neurosteroids (Mody and Pearce, 2004).

Barbiturates (e.g., thiopental) also allosterically potentiate GABA_A_Rs at clinically delivered doses. Evidence suggest sites of action within α and β subunits, and potentially elsewhere. Hippocampal CA1 phasic currents appear to be more sensitive than tonic to potentiation by thiopental (Bieda et al., 2009), although significant effects were seen on both. The largely cerebellar α6 extrasynaptic receptor has a uniquely high sensitivity to pentobarbital (Drafts and Fisher, 2006), perhaps underlying anticonvulsant and ataxic properties, although potentiating effects can also be seen on α4β2γ2 and α4β2δ (Akk et al., 2004). Of historical note, the barbiturates were once the mainstay class of anesthetic agents. With the exception of thiopental, they were largely replaced for routine anesthesia due to a range of undesirable side-effects including the potentially dangerous overlap between anesthetic range and lethal doses. However, they remain in use for veterinary euthanasia (e.g., for biomedical research animals) and for capital punishment via lethal injection.

## Specific Subunit Compositions As Neuroanatomical Targets of Anesthesia

The exact receptor subtypes that underlie tonic and phasic currents are particular to brain region. Here we summarize receptor subtype distribution, and for each receptor population its hypothesized role in anesthesia. For this review, we focus more on regions of the brain heavily involved in consciousness and memory, to reflect clinical concerns about depth of anesthesia and lingering effects on attention and memory observed.

### The Hippocampus

Traditionally associated with the formation of memories, the hippocampus has been studied for decades in order to understand anesthetic mechanisms in a convenient, standardized laboratory model, specifically the acutely prepared rodent *ex vivo* brain slice. From this paradigm, we have considerable knowledge of cellular substrates that mediate anesthetic amnesia and potentially the long-lasting effects of anesthetics on memory function. Within hippocampus CA1-3 subfield pyramidal neurons, phasic current is mediated by mostly α1β2/3γ2 intrasynaptic receptors (Sperk et al., 1997; Caraiscos et al., 2004a), and tonic current by α5β3γ2 extrasynaptic receptors (Bonin et al., 2007). Within the dentate gyrus (DG), which controls afferent input into the CA regions, principal granular cell neurons phasic current is mediated by α1β2/3γ2 intrasynaptic receptors, whereas tonic current is mediated instead largely by α4βxδ extrasynaptic receptors, with some additional tonic conductance likely from α5β3γ2s as well (Herd et al., 2008). The amnestic effects of alcohol are thought to be a consequence of δ-subunit mediated sensitivity of these α4βxδ receptors to alcohol (Mody et al., 2007).

The difference between tonic current receptor expression within these subfields may be related to intrinsic differences between CA3/CA1 pyramidal cells and DG granule cells. The DG is located at the entry of entorhinal excitatory projections into the hippocampus, and hypothesized to have an important role in “gating” or filtering the excitatory input into the dorsal CA subfields, which themselves are mostly excitatory neurons with more active phenotypes. In electrophysiologic study DG granule cells are described as a low-excitability reluctantly firing phenotype- defined by the absence of action potentials even in response to high-intensity current injection- owing partially to the strong tonic inhibitory conductances mediated by α4βxδ extra-synaptic receptors and a more hyperpolarized resting membrane potentials compared to CA neurons (Coulter and Carlson, 2007). This strong tonic inhibition is thought to be important for spatial information processing and memory information because a high firing threshold would ensure that only small, selective population is activated within a single context, constraining inappropriate signal transfer into the hippocampus. During spatial navigation, DG granule cells fire sparsely, with greater place-field specificity than CA3 neurons (Jung and McNaughton, 1993; Senzai and Buzsaki, 2017). Based on these properties, the prevailing theoretical function for the DG as a computational entity is pattern separation, or the ability to de-correlate similar inputs and generate dissimilar outputs, which would be essential to create discrete neural representations (Knierim and Neunuebel, 2016). Moreover, limiting excitation has an important protective feature for “gating” the hippocampal network, which is laden with recurrent connections and prone to epileptogenesis (Lothman et al., 1992; Peng et al., 2004). The importance of tonic inhibition for creating a low-excitability cellular phenotype can be further observed in an array of epileptogenic phenotypes where extrasynaptic GABA_A_R receptor-mediated tonic inhibition is compromised (Maguire et al., 2005; Ferando and Mody, 2012; Li et al., 2013).

Within hippocampal interneurons, the effect of modulating tonic inhibition is cell-type specific. For example, almost all fast spiking parvalbumin-expressing interneurons in DG express α4β3δ GABA_A_R –mediated tonic currents, whereas a minority of somatostatin-expressing interneurons and no calbindin or calretinin-expressing do (Milenkovic et al., 2013). This illustrates a general principal that specialized interneuron species have characteristic firing patterns based on particular calcium-binding protein expression (e.g., parvalbumin and calbindin) and GABA_A_R receptor subunit expression. Unsurprisingly given the importance of this interneuron subpopulation in rhythmogenesis, deletion of the β3 extra-synaptic receptor subunit generates abnormalities in hippocampal theta and gamma oscillations (Hentschke et al., 2009). Tonic current-mediated changes in gamma oscillations can be observed as an effect of ovarian-cycle linked changes in α4β3δ expression, perhaps underlying peri-menstrual cognitive side-effects (Barth et al., 2014).

### The Thalamus

Somatosensory information from the external world is integrated in specific thalamic nuclei before complex processing by the cortex. Thus, thalamic neural processes are uniquely important in the control and transformation of conscious experiences, perhaps like a dynamic gate (Steriade, 2000). Anesthetic-induced depression of neuronal activity in thalamic nuclei and subsequent suppression of the thalamo-cortical circuit are thought to underlie loss of consciousness and unresponsiveness to sensory input.

During awake states, subcortical arousal areas depolarize thalamic neurons, which mediate the integration of information in important cortical areas such as the hippocampus and prefrontal cortex (Llinas and Steriade, 2006). During states of impaired consciousness such as drowsiness, anesthesia, or slow-wave sleep, hyperpolarized thalamic relay neurons hyperpolarize and generate slow oscillations that entrain cortical neurons. Typically two slow rhythms result and dominate the non-REM sleep EEG, delta waves and alpha waves. During anesthesia, EEG recordings of fully anesthetized patients typically show either strong slow wave activity or “burst suppression” spectral patterns where high frequency activity is interleaved with periods of very slow or flat-lined “isoelectric” electrical activity (Brown et al., 2010; Chander et al., 2014). These isoelectric EEG epochs, characteristic of comatose brains, are never found in natural sleep patterns and thought to be an effect of very deep anesthesia. The predominant understanding is that these patterns are generated by the effect of anesthetics on extra-synaptic receptor mediated tonic currents in the ventral basal thalamic neurons. These regions are enriched in α4β2δ extra-synaptic receptors. *In vitro*, this subtype is very sensitive to isoflurane (Jia et al., 2008). *In vivo*, firing of thalamo-cortical relay neurons is strongly depressed by isoflurane in a GABA dependent manner (Jia et al., 2005).

The exact contributions of receptor subtypes have been mostly tested with respect to naturally occurring (e.g., sleep) induced oscillatory behaviors, and implications for anesthetic-specific oscillations are currently limited by assumed similarities and a few insightful studies (Steriade, 2000). Evidence currently suggests that thalamocortical oscillations are entrained by phasic inhibition, but largely depend on tonic inhibition to occur (Rovo et al., 2014). Thalamic removal of γ2, causing cell surface depletion of local intrasynaptic receptors, did not abolish slow thalamocortical oscillations or sleep spindles but did alter single-neuron burst firing. Because thalamocortical network activity is essential to consciousness, this would suggest that alterations in GABA_A_R surface expression affect sensitivity to hypnotics.

### The Cortex

The cortex is most associated with anesthetic hypnosis. In most layers the extra-synaptic receptor is α5β3γ2, but in layer 2/3 extra-synaptic receptors demonstrate the α4β2δ configuration. These generalizations about cortical layers should be taken with consideration to the region, because the stratification of cortical layers can have a specific functional role in different processes. Cortical layers 2/3 receive information from other cortical areas. Slow oscillations like delta waves are only detected on EEG when all layers II/III and V are synchronized with thalamic inputs, and otherwise the beta frequency activity dominates (Harrison, 2007). During wakefulness, alpha and beta emanating most strongly from the posterior of the brain, namely the occipital lobe, predominate cortical EEG when the eyes are closed. During induction, alpha decreases globally but there also is a spatial shift or “anteriorization” in alpha power, meaning that it is now strongest from the frontal cortex (Brown et al., 2010; Mashour, 2014). This effect is also thought to be based on the expression of anesthetic sensitivity of α4β2δ receptors on ventrobasal thalamus neurons. These neurons project to the frontal cortex, entraining their activity. In contrast, the posteriorly projecting thalamic nuclei do not express these receptors. This model is supported by functional MRI measurements taken during propofol anesthesia. Resting state network analysis suggests that disconnect between networks within the frontal cortex is part of loss of consciousness by propofol (Guldenmund et al., 2016).

Phasic and tonic currents mediate distinct temporal modulation of single-cell neuronal activity, and contribute differently to the microscopic conductances that generate patterns of neuronal population activity empirically observed from human patients, such as evoked field potential or EEG recordings. A straightforward example can be made of the receptors mediating cortical local inhibition. The most superficial layer of cortex is largely populated by the apical dendrites of layer 5 pyramidal excitatory neurons, and their dendritic local field potentials predominate the forebrain scalp EEG signal (Brown et al., 2010). Here, intrasynaptic α1βxγ2 receptors mediate synaptic inhibition from local interneurons, whereas the extra-synaptic α5β3γ2 receptors mediate tonic inhibition of cortical neuron activity (Ali and Thomson, 2008). Judging the comparative contribution to the EEG signal is less straightforward; because the α5β3γ2 receptors are heavily expressed in the distal dendrites whereas the α1βxγ2 receptors are somatically expressed (Sur et al., 1999b), the extra-synaptic receptors are more superficial and may arguably better represented in the scalp EEG. These distinctions may be important for interpreting the effects of subtype-specific pharmacology. Of course, these various inhibitory inputs converge upon synaptic integration, and ultimately dendritic field potentials are a biased reflection of cortical activity.

Sedation is also thought to arise from anesthetic effects on cortical systems. Although qualitatively akin to a mild hypnosis, there is evidence for molecular targets that are more specific for sedation. For example, etomidate-mediated sedation but not hypnosis is lost in the β2(N265S) transgenic mouse, and the action of the benzodiazepine diazepam at α1His101 provides sedation but not hypnosis (Bonin and Orser, 2008).

### Subcortical Arousal Centers

Wakefulness and somnolence are governed by a system of neuromodulatory projection neurons that originate in several subcortical nuclei that widely innervate the brain. Rationally, anesthetic action on these regions is a clear and direct mechanism for loss of consciousness. However, predicting the contribution of these subcortical arousal centers to anesthesia is complicated by the involvement of these systems in sleep, as well as redundant arousal circuitry. When, GABA_A_R current sensitivity to propofol was tested in three arousal centers targeted by natural sleep pathways using the β3(N265M) transgenic mouse, the perifornicate and tuberomamillary nucleus (TMN) but not the locus coeruleus were affected (Zecharia et al., 2009). TMN is in hypothalamus, projects the arousal neurotransmitter histamine into cortex to generate wakefulness. In anesthesia, propofol enhancement of GABA in TMN depresses histamine release into the cortex (Zecharia and Franks, 2009). Excellent and thorough reviews of subcortical areas involved in arousal and their influence on sleep and anesthesia can be found elsewhere (Brown et al., 2011).

### Spinal Cord and Peripheral Nervous System

Early seminal experiments demonstrated that distinct anesthetic targets within the brain and spinal cord mediate distinct mechanisms for hypnosis vs. analgesia, respectively (Antognini and Schwartz, 1993; Antognini and Carstens, 1998; Antognini et al., 1998). The classical molecular sites for anesthetic analgesia, or blockade of nociception, lie in the spinal cord dorsal horn, where primary afferents innervating somatic and visceral tissue first synapse onto CNS neurons to relay sensory information. Anesthetic potentiation of GABA_A_Rs on pain-mediating nociceptive afferent presynaptic terminals as well as GABA_A_Rs on post-synaptic neurons largely in lamina II/substantia gelatinosa contribute to blunted relay of sensory information. Evidence suggests these are largely α2/3β2/3γ2 intrasynaptic subtypes (Bohlhalter et al., 1996). Anesthetic actions on GABA_A_Rs within the ventral horn (intrinsic neurons and motor neurons) contribute to anesthetic immobility and muscle relaxation, although during surgery adjunct drugs acting directly on the neuromuscular junction are typically given to reinforce these effects.

## GABA_A_R Surface Expression is Controlled by Intracellular Trafficking

The strength of inhibitory currents, tonic or phasic, depends on the number of receptors expressed on the neuronal cell surface. This number is dynamically maintained because GABA_A_Rs undergo controlled rates of endocytosis, exocytosis, and degradation- in effect constantly recycling between the surface and the cytoplasm. These events are coordinated by a myriad of effector and regulatory proteins, themselves controlled by second messengers signaling events. Perturbations to all of these steps can potentially alter GABAergic signaling, e.g., an increase in surface expression and current can arise from increased rates of exocytosis, reduced endocytosis, or decreased degradation. Given that changes in surface expression can be observed *in vitro* on the time course of 15–30 min, it is conceivable that routine anesthesia care has major influence on the dynamics of membrane surface expression. A basic review of fundamental trafficking mechanisms is presented first, before a discussion of mechanisms for pharmacologic and activity based changes in GABA_A_R surface expression.

## Receptor Biosynthesis, Exocytosis, and Subcellular Localization

GABA_A_ receptor subunits are synthesized in the endoplasmic reticulum (ER). Correctly oligomerized channels under post-translational processing within the Golgi apparatus and then actively exported to the cell surface and inserted into the plasma membrane by exocytosis (Luscher et al., 2011). Imaging and biochemical assays suggest that receptor insertion occurs extra-synaptically (Bogdanov et al., 2006), and intrasynaptic receptors undergo additional targeting to the post-synaptic density via lateral diffusion (Bannai et al., 2009). Kinetic studies suggest that movement into the synapse happens quickly, as the ratio of synaptic/extra-synaptic β3s increases significantly within 15 min (Bogdanov et al., 2006). Synaptic confinement involves direct interaction gephyrin (Jacob et al., 2005), the cytoskeletal-bound post-synaptic scaffolding protein of inhibitory synapses, through binding sites on α1, α2, α3, β2, and β3 subunit large cytoplasmic loops (Tretter et al., 2012).

Keeping extra-synaptic receptors in place is less understood. The α5β3γ2 receptors affix to the cytoskeleton in extra-synaptic clusters by the scaffolding protein radixin. Their extra-synaptic confinement is not static, and dissociation from radixin and into synapses has been identified as a novel mechanism means of synaptic strengthening (Hausrat et al., 2015). Within synapses, α5β3γ2 receptors mediate a slowly decaying component of IPSCs through transient interactions with gephyrin (Brady and Jacob, 2015). GABA_A_R exchange between intrasynaptic and extra-synaptic sites is under active investigation as a potentially important form of inhibitory plasticity (Triller and Choquet, 2005). The extra-synaptic location of α4βxδ receptors is attributed to absence of a gephyrin binding site within the α4 protein sequence, as an extra-synaptic scaffolding protein for α4βxδ has not been identified. In general, structural and regulatory elements for extra-synaptic receptor densities have not been well characterized. Because of their unique pharmacological role in anesthetic-hyperpolarization, understanding the mechanics of extra-synaptic receptor movement within the cell and its surface is a critical knowledge gap in anesthetic mechanisms.

## Endocytosis and Post-Endocytotic Sorting: Recycling Vs. Degradation

GABA_A_Rs are constantly removed from the cell surface via clathrin-mediated endocytosis, which is orchestrated by complex sequences of proteins interactions all critically dependant on subunit-specific endocytotic regulatory site. These physical interactions are subject to modulation by post-translational modifications (especially phosphorylation), and aberrations can dramatically affect surface expression. The details are well-summarized elsewhere (Nakamura et al., 2015). Because these regulatory processes depend on specific protein residues, anatomical and behavioral phenotypes can be observed in a subunit-specific manner. For example, transgenic knock-in mice expressing γ2 (Y265/7F) phosphomimetic receptors, which lack γ2 Y265/7 mediated endocytosis, have increased γ2 cell surface expression, larger inhibitory synapses, and impaired spatial memory (Kretschmannova et al., 2013). Notably, this trafficking phenotype engenders an abnormal responses to anesthesia, as female γ2(Y256/7F) mice are more sensitive to the amnestic and hypnotic effects of propofol. The basis is likely the higher measurements of α4βxδ in the thalamus and DG as a result of compensatory changes in gene expression and surface trafficking that is gender-sensitive. Induced changes endocytotic regulation may have a rapid effect on surface expression because of the relatively quick rates of constitutive intrasynaptic receptor endocytosis. Biochemical rate-measurements suggest that intrasynaptic receptor constitutive endocytosis occurs rapidly, with approximately 20–25% of surface receptor β3 subunits internalizing by 30 min in neuron cultures (Kittler et al., 2004). Other groups have shown significant internalized γ2 protein after 15 min (Joshi and Kapur, 2009).

Likewise, specific endocytotic mechanisms for the α4βxδ receptors mediating tonic current are mediated through unique sequences on the δ subunit (Gonzalez et al., 2012). Kinetically, constitutive endocytosis of δ-containing receptors appears to occur more slowly than intrasynaptic receptors. The surface half-life of δ subunits is 103–171 min depending on the measurement method, immunostaining in culture or slice biotinylation in organotypic hippocampal slices, respectively (Joshi and Kapur, 2009). However, ethanol-evoked endocytosis of α4βxδ happens more quickly, as significantly diminished surface expression can be measured within 15 min (Gonzalez et al., 2012). Given the role of α4βxδ-mediated tonic current in thalamocortical and hippocampal circuits underlying anesthetic hypnosis and amnesia, α4βxδ trafficking changes have a direct bearing on the behavioral response to anesthesia. For example, latency to loss of righting reflex (metric for sensitivity to anesthetic hypnosis) increases 12 and 24 h following ethanol intoxication, suggesting a desensitization to isoflurane (Liang et al., 2007).

Once internalized, GABA_A_R containing endosomes are sorted between recycling endosomes or late endosomes destined for lysosomal degradation. Recycled receptor endosomes reside transiently beneath the cell surface. Intracellular GABA_A_Rs residing near gephyrin underneath the synapse have been identified, presumably forming receptor reservoirs that can be rapidly mobilized to mediate short-term inhibitory synaptic plasticity (van Rijnsoever et al., 2005).

Ubiquitinylated receptors can also be targeted for degradation within lysosomes, acidic organelles which enzymatically digest the proteins inside. Again, this process appears to happen relatively quickly, as rate studies in cultured neurons suggested that 30% of neuronal GABA_A_Rs degraded over the course of 6 h (Kittler et al., 2004). Overall, it is clear that GABA_A_R intracellular movement is a complicated dynamic process with rapid kinetics, and very subtle changes in protein signaling and have the capacity to change surface expression and phenotype very quickly.

## Anesthetics and GABA_A_R Surface Expression

Recent research has shown that anesthesia itself may cause changes to the surface expression of GABA_A_Rs and other ion channels that control neurological function. At present, these changes appear to be mediated by the effect of anesthetics on the intracellular signaling pathways that control trafficking. Propofol was demonstrated to increase β3 subunit surface expression in hippocampal brain and primary cultures within 15, 30, and 60 min of exposure (Li et al., 2015). The underlying mechanism is PKC-mediated phosphorylation of β3 at the AP-2 binding domain. Immunoprecipitation of β3 from hippocampal lysates show a depressed association with AP-2 subunit β-adaptin, suggesting diminished recruitment of the entire AP-2 complex. Surface expression of GluR1, a subunit of AMPA receptors which are also trafficked using AP-2 and clathrin-mediated endocytosis, was not significantly affected, suggesting that propofol is not impairing general endocytotic mechanisms. Activated PKCε is increased at these timepoints. PKCε kinase activity on β3 receptors measured with hippocampal lysate and recombinant PKC shows increased β3 phosphorylation. The change in surface expression and decrease in AP-2 subunit binding are blocked with PKCε inhibitor (Li et al., 2015). Functionally, propofol causes an increase in evoked and miniature IPSC amplitude that can be partially reversed with PKCε inhibitor. Post-translational modification of channel kinetics by PKC and allosteric enhancement of channel function by propofol likely contribute to this effect, but the effect of the inhibitor suggests that ultimately propofol is creating a temporary sensitization of GABAergic transmission through PKCε-dependant mechanisms. Similarly, a temporary increase in α5 surface expression has been associated with etomidate administration (Zurek et al., 2014). This increase appears to be in part responsible for post-anesthesia memory impairment. The expression levels return to normal in 2 weeks.

Several networks of second messengers and signaling protein regulators that control or influence the subcellular movement of GABA_A_Rs are also potentially sensitive to anesthetics. The interactions of these pathways with drugs commonly used in anesthesia are just being recognized and remain an area of active investigation. Here, we briefly introduce canonical pathways known to impact GABA_A_R surface expression, describe the basis for anesthetic-sensitivity, and speculate hypothetical impact on GABA_A_R surface expression.

### Calcium and IP3R Pathway

Calcium is one of the most important intracellular second messengers, and isoflurane itself will raise intracellular calcium by directly activating the inositol 3-phosphate receptor (IP3R) channel resulting in the release of stored calcium into the cytosol (Wei and Xie, 2009). Several proteins regulating of GABA_A_R surface expression are calcium-dependent, including PKC which controls trafficking of several subunits through many phosphorylation sites (Abramian et al., 2010; Nakamura et al., 2015).

### PKC

Propofol itself can activate and allosterically enhance PKCε, a calcium-independent isozyme. *In vitro* studies of the direct interaction between propofol and the regulatory domain of PKC promotes autophosphorylation at Ser729, a final step in initiating and maintaining enzyme activation. In addition to the propofol-mediated surface β3 discussed earlier (Li et al., 2015), the activation of PKCε by propofol also leads to downstream activation of the transcription factor target CREB (Wickley et al., 2009). The transcriptional control of α1 surface expression by PKC/CREB signaling has already been demonstrated (Hu et al., 2008), but not testing following propofol.

### Rho Kinase/ROCK Signaling

Propofol has been shown to interfere with neuronal development by causing neurite retraction through the Rho kinase/ROCK signaling pathway (Bjornstrom et al., 2014), through effects on PKCε. This interaction may be significant for surface expression because Rho/ROCK signaling regulates α5β3γ2 extra-synaptic localization by controlling α5-radixin binding, which in turn affects synaptic α5β3γ2 levels (Hausrat et al., 2015).

### Inflammatory Signaling

The interaction between volatile anesthetics and the endogenous neuroimmune system is under great scrutiny as a factor in the development of cognitive impairments following surgery, such as post-operative delirium and post-operative cognitive dysfunction (Vutskits and Xie, 2016). One important line of reason is the relationship between inflammation and increased extra-synaptic GABA_A_Rs surface expression and associated memory impairments. Application of the inflammatory cytokine IL-1β to hippocampal neurons increases α5β2γ2 surface expression, increasing both tonic current and its potentiation by anesthetics (Wang et al., 2012; Avramescu et al., 2016). Mice treated with lipopolysaccharide injections, which models sepsis by stimulating the innate immune system, show potentiated hypnosis and ataxia from isoflurane and etomidate (Avramescu et al., 2016). Depressions in spatial memory and fear based learning, and long-term potentiation seen in these animals appears to be reversible with α5 receptor antagonist, α5 subunit deletion, or blockade of p38 mitogen-activated protein kinase signaling (Wang et al., 2012). Unsurprising, septic patients display anesthetic hypersensitivity (Monk et al., 2005). We speculate that blockade of inflammatory signaling in the brain, either that induced by volatile anesthetics themselves and/or those induced by systemic inflammation, may protect against post-operative neurocognitive impairments partially by preventing the development of deleterious changes in GABA_A_R surface expression.

## Summary

The perioperative experience is an opportunity for profound dynamic changes in GABA_A_R expression. Despite specific knowledge regarding individual subunit compositions subcellular localization, regional anatomy, and pharmacological attributes, we still lack understanding of the contribution of these specifics to intra- and post-operative consequences. Off-target effects on receptor trafficking subtype expression can impart neurophysiologic differences that likely influence the heterogeneity in behavioral endpoints of anesthesia, and variability in cognitive recovery trajectories following emergence. Because of the profound differences in GABA-signaling between developing and mature neurons – a focus on experiments using mature neurons should be emphasized, despite the increased technical challenges of working with older neurons *in vitro*. Future research avenues include studies using combined living cell microscopy and photolabeling that can help detect important changes in expression levels on relevant time scales.

General anesthetics are invaluable and unique among medicines because of their capacity for controlled manipulation of consciousness. Although substantial work has been done to characterize the effect of anesthetic agents on biophysical function of GABA_A_Rs and the resultant effect on global patterns of brain activity, much less is known about the long-term consequences on neurological function. Experimental studies increasingly show that anesthetic agents have the potential to induce persistent changes in neurophysiology, which has serious implications for basic and clinical science alike. A comprehensive understanding of these processes will be necessary for predicting how persistent adverse effects in neurological function arise and determining what factors underlie resilience to the effects of anesthetics.

## Author Contributions

IS performed the literature search, organized the theme, wrote the first draft, and constructed the table. EB and PG added key concepts/references as well as co-wrote and co-edited the manuscript.

## Conflict of Interest Statement

The authors declare that the research was conducted in the absence of any commercial or financial relationships that could be construed as a potential conflict of interest.
